# Pilot-Scale Assessment of Urea as a Chemical Cleaning Agent for Biofouling Control in Spiral-Wound Reverse Osmosis Membrane Elements

**DOI:** 10.3390/membranes9090117

**Published:** 2019-09-06

**Authors:** Huma Sanawar, Szilárd S. Bucs, Martin A. Pot, Jure Zlopasa, Nadia M. Farhat, Geert-Jan Witkamp, Joop C. Kruithof, Mark C. M. van Loosdrecht, Johannes S. Vrouwenvelder

**Affiliations:** 1 King Abdullah University of Science and Technology (KAUST), Water Desalination and Reuse Center (WDRC), Division of Biological and Environmental Science and Engineering (BESE), Thuwal 23955-6900, Saudi Arabia; 2 Evides Industriewater BV, Process & Technology Department, P.O. Box 4472, 3006 AL Rotterdam, The Netherlands; 3 Department of Biotechnology, Faculty of Applied Sciences, Delft University of Technology, Van der Maasweg 9, 2629 HZ Delft, The Netherlands; 4 Wetsus, European Centre of Excellence for Sustainable Water Technology, Oostergoweg 9, 8911 MA Leeuwarden, The Netherlands

**Keywords:** desalination, urea, biofouling, chemical cleaning, nanofiltration, membrane autopsy

## Abstract

Routine chemical cleaning with the combined use of sodium hydroxide (NaOH) and hydrochloric acid (HCl) is carried out as a means of biofouling control in reverse osmosis (RO) membranes. The novelty of the research presented herein is in the application of urea, instead of NaOH, as a chemical cleaning agent to full-scale spiral-wound RO membrane elements. A comparative study was carried out at a pilot-scale facility at the Evides Industriewater DECO water treatment plant in the Netherlands. Three fouled 8-inch diameter membrane modules were harvested from the lead position of one of the full-scale RO units treating membrane bioreactor (MBR) permeate. One membrane module was not cleaned and was assessed as the control. The second membrane module was cleaned by the standard alkali/acid cleaning protocol. The third membrane module was cleaned with concentrated urea solution followed by acid rinse. The results showed that urea cleaning is as effective as the conventional chemical cleaning with regards to restoring the normalized feed channel pressure drop, and more effective in terms of (i) improving membrane permeability, and (ii) solubilizing organic foulants and the subsequent removal of the surface fouling layer. Higher biomass removal by urea cleaning was also indicated by the fact that the total organic carbon (TOC) content in the HCl rinse solution post-urea-cleaning was an order of magnitude greater than in the HCl rinse after standard cleaning. Further optimization of urea-based membrane cleaning protocols and urea recovery and/or waste treatment methods is proposed for full-scale applications.

## 1. Introduction

Membrane filtration processes are regarded as a solution to overcome freshwater scarcity by enabling the utilization of water sources such as seawater and wastewater to produce clean water for industrial, agricultural, municipal and potable use. Reverse osmosis (RO) is by far the most commonly applied membrane filtration process due to its effectiveness in removing pollutants and monovalent ions, ease of operation and cost-effectiveness [1,2]. The use of polyamide RO membranes in spiral-wound configurations is prevalent in commercial systems [3]. The fouling of membrane modules continues to be the major limitation in the effective application of RO membrane installations. More than 45% of all cases of membrane fouling are caused by biofouling (excessive growth of biomass) [4] leading to filtration process failure, high operational costs and early membrane replacement. Biofouling is operationally diagnosed when the normalized pressure drop (NPD) is increased by 15% or the normalized flux is decreased by 10% of the start-up values [5,6,7].

Biofouling is inevitable during long-term operation [8]. Even with extensive pretreatment of feed water using disinfectants [9] and biocides [10], it is not possible to remove all micro-organisms and biodegradable substances from the feed water [11,12]. Membrane surface and feed spacer modifications can somewhat delay biofilm formation [13,14,15], but cannot completely eliminate biofouling. However, slowing down the biofilm formation rate gives plant operators more time to diagnose the cause of biofouling and implement the most appropriate control strategies. Bucs et al. (2018) recommended that research should be aimed at (i) slowing down biofilm formation; (ii) reducing its impact on membrane performance and (iii) removing biofilms by advanced cleaning strategies. The work presented herein pursues an advanced cleaning strategy for biofouling control.

Chemical cleaning of membrane modules is routinely carried out at full-scale installations to restore membrane performance. Several studies have highlighted the need for new chemical cleaning strategies since the conventional cleaning by NaOH and HCl fails to provide highly effective membrane regeneration [16,17,18,19]. The ability of urea to dissolve biofilm proteins has been discussed briefly in the literature [20,21,22]. Recently, controlled laboratory experiments have been conducted using membrane fouling simulators to assess the efficiency of concentrated urea as a chemical cleaning agent for RO membranes [23]. These studies demonstrated considerable potential of urea to enhance biofilm solubilization and removal. However, lab-scale biofouling experiments may not be completely representative for “real world” membrane applications whereby a combination of biofouling, colloidal fouling, inorganic scaling and the organic fouling of membranes may occur simultaneously. Membrane performance at full-scale RO plants is also affected by variations in feed water parameters, pre-treatment techniques and their effectiveness, as well as operating conditions (such as flux) [24]. Membrane autopsies of full-scale modules are therefore necessary in order to (i) identify the types and degree of fouling, and (ii) ascertain the applicability of novel chemical cleaning agents and strategies at full-scale installations.

This study examined the efficiency of urea as a chemical cleaning agent in contrast to the conventional acid/alkali cleaning protocol, applied to 8-inch diameter spiral-wound RO membrane elements taken from a full-scale installation and cleaned in a pilot test facility. To fulfil this objective, performance data of the membrane modules was assessed and a subsequent autopsy of the membrane modules was carried out, enabling detailed laboratory analysis using advanced analytical techniques which had not been incorporated in our previous lab-scale studies [23].

## 2. Materials and Methods

### 2.1. Experimental Design

The pilot test facility was located at the DECO water treatment plant, managed by Evides Industriewater in Terneuzen, the Netherlands. The DECO plant produces demineralized water and cooling tower supply water from membrane bioreactor (MBR) permeate, originating from the wastewater treatment plant of Terneuzen [9]. The MBR permeate is transported over 13 km by pipeline to the DECO plant. Monochloramine is dosed in the pipeline to prevent biofouling. At the DECO plant, the effluent is passed through a 50 µm automatic screen filter. The residual monochloramine is removed by dosing a small excess of sodium hydrogen sulfite just upstream of the RO. The average flow to the RO is 254 m^3^/h. The system consists of two-pass RO units containing more than 700 8-inch FILMTEC™ membrane elements (BW30XFR-400/34i) (Delfgauw, the Netherlands) comprising 34 mil feed spacers. The production design capacity of each RO unit is 150–175 m^3^/h. At the plant, membrane fouling problems are inevitable due to the high fouling tendency of wastewater effluent. The decline in plant performance is identified as a rapid increase of feed channel pressure drop, requiring preventive cleaning as well as regular maintenance cleaning (using NaOH and HCl) every 3 days to avoid irreversible loss in membrane performance.

For this study, an automatic RO pilot installation equipped with two single modules 8-inch pressure vessels was employed, built by Logisticon Water Treatment (Groot-Ammers, the Netherlands) (Supplementary Material Figure S1). Freshwater (surface water from Belgium) was sourced as feed water to the RO pilot installation. Three fouled 8-inch membrane modules of the same age of operation (2.5 years) were harvested from the lead position of one of the full-scale RO units treating MBR permeate. Each spiral-wound membrane element had a surface area of 37.16 m^2^ (or 400 sq. ft.) and was mostly fouled on the inlet side (Supplementary Material Figure S2).

A membrane element was placed inside a vertically installed cleaning-in-place (CIP) vessel and feed water was fed from the top (inlet side of the module). The pilot installation was designed to record three key performance indicators (KPIs) including pressure, flow and conductivity. With the freshwater feed, the initial performance of the membrane element—including feed and concentrate flow (m^3^/h) and pressure (bar)—was measured. Second, the module was rinsed with demineralized water twice to displace the feed water. The demineralized water was drained from the system to avoid dilution of the chemical cleaning solutions before proceeding with the cleaning protocol. Table 1 describes the chemical cleaning protocol applied to each of the three membrane modules. NaOH and HCl were obtained from the DECO water treatment plant’s chemical storage unit. Urea as crystals (technical grade, uncoated) was obtained from the chemical supplier for DECO (Brenntag Nederland BV, Dordrecht, the Netherlands).

Demineralized water was used as the solvent to prepare 100 L of the cleaning solutions. The cleaning solutions (NaOH and urea) were heated to 35 °C because this is the optimum temperature for NaOH/HCl cleaning aimed at enhanced flux recovery [25]. The alkali cleaning was applied at a concentration typically used in industry for chemical cleaning (0.01 M NaOH) and is reportedly quite effective at restoring membrane performance [7,26]. The performance of the membrane elements was assessed after the recirculation of the first cleaning agent (NaOH or urea) and at the end of the cleaning protocol. After cleaning, membrane elements were stored and transported to the laboratory in heavy duty plastic bags containing ice for autopsy, sampling and analysis.

### 2.2. Key Performance Indicators

Membrane fouling results in an increase in pressure drop across the feed channel and a decline in permeate flux. Monitoring these KPIs allows for the early identification of fouling and is used to determine the cleaning frequency and strategy. The normalization of the performance indicators is required in order to accurately assess and compare the process performance, independent of varying parameters such as feed water temperature and flow. In this study, pressure drop was normalized for flow and temperature using the same method (see Equation 1) as employed by the DECO plant [27].

(1) $$NPD=\;\Delta P_{ACT}.Q_{CF\Delta P}.T_{CF\Delta P}=\left(P_{FEED}-P_{CONCENTRATE}\right){\left(\frac{\left(Q_{FEED,REF}+Q_{CONCENTRATE,REF}\right)\;÷2}{\left(Q_{FEED}+Q_{CONCENTRATE}\right)\;÷2}\;\right)}^{1.6}.{\left(\frac{\eta_{T,REF}}{\eta_{T,ACT}}\right)}^{0.4}$$

where *NPD* is the normalized pressure drop (bar), ΔP_ACT_ is the actual pressure difference, *Q_CF,ΔP_* is the flow correction factor, *T_CF,ΔP_* is the temperature correction factor, *P_FEED_* is the feed pressure, *P_CONCENTRATE_* is the concentrate pressure, *Q_FEED,REF_* is the reference feed flow rate (m^3^/h), *Q_CONCENTRATE,REF_* is the reference concentrate flow rate, *Q_FEED_* is the feed flow rate, *Q_CONCENTRATE_* is the concentrate flow rate, *η_T_,_REF_* is the viscosity (Pa·s) at reference temperature (°C) and *η_T,ACT_* is the viscosity at actual water temperature.

Flux (permeation rate) is defined as the water volume flowing through the membrane per unit area and time (Lm^−2^ h^−1^) [28]. The water flux normally increases by 3% for each degree of water temperature increase. Therefore, normalization of the flux to a standard temperature of 25 °C (for RO/NF membranes) accounts for fluctuations in water viscosity, as shown in Equation (2).

(2) $$J_{25}=\left(Q_{PERMEATE}\;÷\;A_{MEMBRANE}\right)\times TCF$$

where *J*_25_ is the permeate flux normalized to a temperature of 25 °C, *Q_PERMEATE_* is the permeate flow (m^3^/h), *A_MEMBRANE_* is the surface area of the membrane module (m^2^), and *TCF* is the temperature correction factor.

### 2.3. Membrane Autopsy

Membrane autopsies were carried out in order to retrieve membrane and feed spacer samples for the qualitative and quantitative analysis of the fouling layer. An electric saw was used to cut off the endcaps of the membrane element and to cut open the fiberglass casing. After the fiberglass casing was removed, the membrane and spacer sheets were unwound and laid out on a clean table. First, visual observations of membrane fouling were made and photographs were taken of the membrane/spacer surface.

Membrane and spacer coupons were acquired from the inlet (most fouled part) of the membrane element. The coupon dimensions were measured with calipers so that the results are reported per area of the combined membrane and spacer surface area. The amount, distribution and composition of the fouling layer were analyzed using various analytical techniques that are described in the following sections.

### 2.4. Biomass Quantification

#### 2.4.1. Adenosine Triphosphate (ATP)

ATP analysis measures the viable biomass content and it is an indicative parameter of biofouling [18,29,30]. Membrane and spacer surface area (3–5 cm^2^) was swabbed with 3M clean-trace surface ATP swabs (3M, Delft, the Netherlands) from the inlet, middle and outlet of the membrane module. The amount of active biomass was measured using the 3M kit according to manufacturer protocol. The results were obtained in relative light units (RLU) and converted to pg ATP/cm^2^ using the equation of line from a calibration curve.

#### 2.4.2. Total Organic Carbon (TOC)

The total carbon content of the accumulated organic matter was determined using total organic carbon (TOC), which is commonly used to obtain information on the degree of biofouling [29,31]. Membrane and spacer coupons (4–9 cm^2^) were cut from the membrane element and placed in centrifuge tubes containing 30 mL of ultrapure water. The tubes were placed in an ultrasonic water bath (Bransonic, model 5510E-DTH, CT, USA, output 135 W, 42 kHz) for two minutes, followed by mixing on a Vortex for one minute to remove biomass from the membrane and spacer surface. The procedure was repeated three times and the coupons were removed from the solution. Since the samples could not be homogenized due to the presence of thick particulate matter, centrifugation was necessary in order to spin down the sediments to a pellet. TOC was measured using a Shimadzu (Japan) TOC-L analyzer and the results were given in mg TOC per cm^2^ of combined membrane and spacer surface area.

The TOC content in the final stage rinse solution (HCl) was measured before and after cleaning the membrane modules. The HCl solution sampled after rinsing the membrane module cleaned with urea was dialyzed over a 3.5 kDa membrane in order to prevent interferences from urea molecules. A volume of 50 mL of the HCl solution post-cleaning was dialyzed over 1000 mL of demi-water. The dialysate solution (demi-water) was replaced two more times over a duration of approximately 30 h. This was to ensure that interferences from urea, if any, would be negligible.

### 2.5. Biofilm Composition

#### 2.5.1. SEM–EDX Analysis

Scanning electron microscopy (SEM) combined with energy dispersive X-ray (EDX) analysis was used to study the elemental composition of the fouled membrane surface. Membrane coupons (4 cm^2^) were acquired from the inlet of each of the membrane modules and air dried. The membrane coupons were cut into smaller sections (about 1 cm^2^) and mounted onto an aluminum stub with carbon tape. The samples were coated with 5 nm iridium inside the Q150T S sputter coater (Quorum Technologies, Lewes, UK). Each sample was examined with SEM (Teneo VS SEM, Thermo Fisher Scientific, Waltham, MA, USA) at two different locations under magnifications ranging from 250× to 25,000×. EDX analysis (Octane Pro EDAX, AMETEK, MA, USA) was carried out on three different locations (full field view), followed by spot analysis at three different spots within each field of view (a total of 12 random locations on each membrane sample). A continuous X-ray energy spectrum from 0 to 10 keV was integrated for each elemental scan. Each element composition value was expressed by the average of three measurements from the full field view analysis for each sample.

#### 2.5.2. ATR–FTIR Analysis

Attenuated total reflection–Fourier transform infrared spectroscopy (ATR–FTIR) was used to examine the molecular composition of the fouled membrane surface. The FTIR instrument (Nicolet is10, Thermo Fisher Scientific, Waltham, MA, USA) contained a SmartiTR diamond ATR accessory (angle of incidence of 45°), coupled with OMNIC software. Membrane coupons (4 cm^2^) were cut from the inlet of each of the membrane modules. After obtaining a blank spectrum (using air as the background signal), the air-dried membrane samples were placed on the sample holder and IR spectra were collected in the spectral range of 4000–525 cm^−1^ with a resolution of 4 cm^−1^ from 32 scans per measurement.

## 3. Results

### 3.1. Membrane Performance

The performance of the membrane modules was characterized by the normalized pressure drop and normalized flux before, during and after cleaning (Table 2).

**Figure 1:**
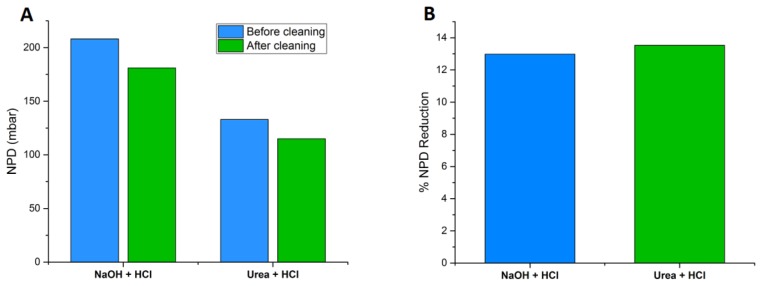
(**A**) Normalized pressure drop (NPD) before and after cleaning and (**B**) percent reduction in NPD after cleaning the reference module (NaOH + HCl) and the test module (Urea + HCl).

The reference module had a normalized pressure drop (NPD) of 208 mbar before cleaning, which was reduced by NaOH/HCl cleaning to 181 mbar (Figure 1A). The NPD for the test module was reduced from 133 mbar before cleaning to 115 mbar after cleaning with urea/HCl (Figure 1A). Thus, both cleaning protocols resulted in a 13% reduction in NPD (Figure 1B). The percent decrease in NPD is calculated from the initial and final values for each membrane module. Therefore, the comparison in percent decrease in NPD after each chemical cleaning is valid because each module is assessed according to its own performance parameters, independent of the parameters of the other two membrane modules.

**Figure 2:**
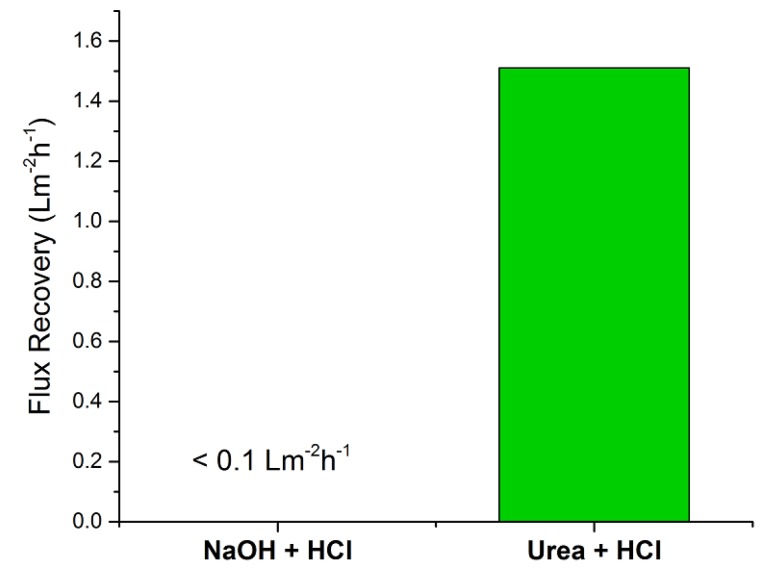
Increase in normalized permeate flux after cleaning the reference module with NaOH + HCl and the test module with urea + HCl.

For the reference module, the normalized permeate flux remained unchanged (29 Lm^−2^ h^−1^) after cleaning with NaOH + HCl. However, cleaning the test module with urea + HCl increased the normalized permeate flux by 1.51 Lm^−2^ h^−1^ (Figure 2).

### 3.2. Visual Analysis

**Figure 3:**
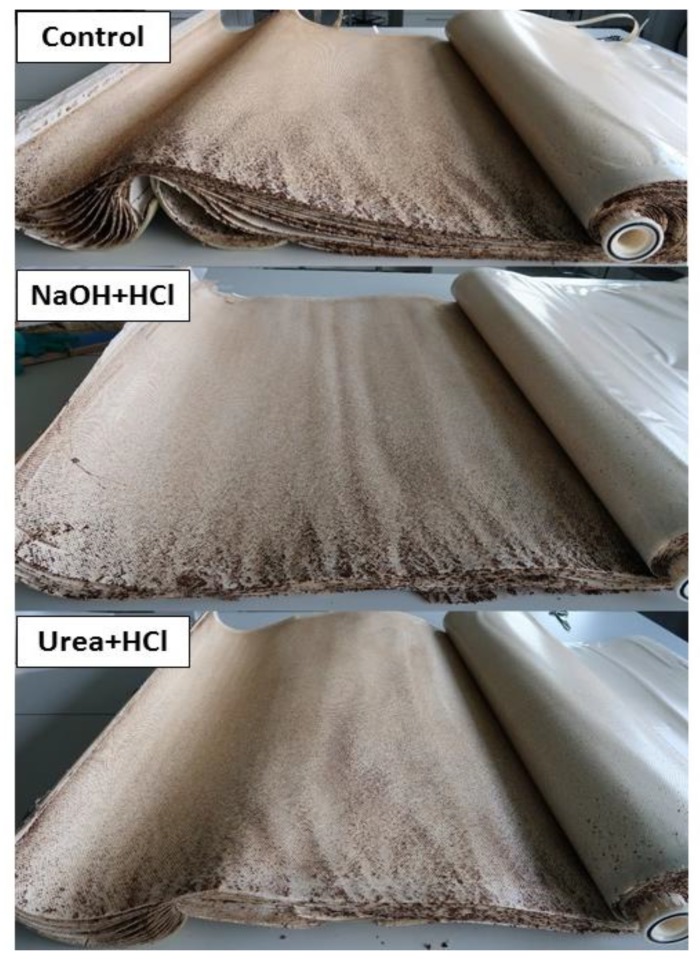
Visual comparison of the membrane/spacer surface of uncleaned (control) and cleaned membrane modules.

An autopsy of the membrane modules confirmed that fouling was predominantly present on the spacer surface at the inlet side of the element (Supplementary Material Figure S3). The membrane module cleaned with urea appeared visibly cleaner compared to the control and reference membrane modules (Figure 3). A close-up view of the control, reference and urea membrane modules is also shown in Supplementary Material Figure S4.

### 3.3. Biomass Parameters

**Figure 4:**
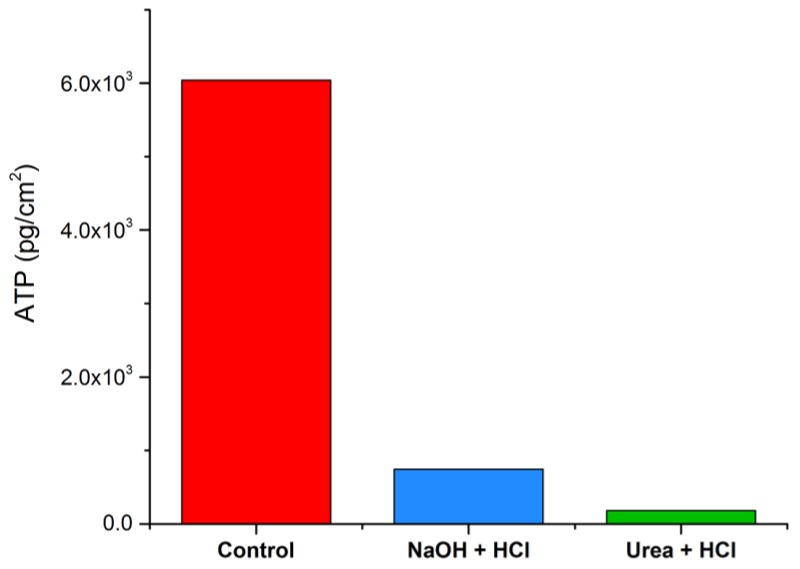
Concentration of active biomass measured as pg ATP/cm^2^ in the control (uncleaned) and cleaned membrane modules.

The concentration of accumulated biomass was quantified using ATP (active biomass parameter). The results of the biomass parameters are presented in Figure 4. The control membrane module (uncleaned) contained the highest amount of active biomass (6.0 × 10^3^ pg ATP/cm^2^). Of the two cleaned membrane modules, the test module (cleaned with Urea + HCl) contained the least amount of active biomass (1.8 × 10^2^ pg ATP/cm^2^) compared to the reference module cleaned with NaOH + HCl (7.5 × 10^2^ pg ATP/cm^2^).

**Figure 5:**
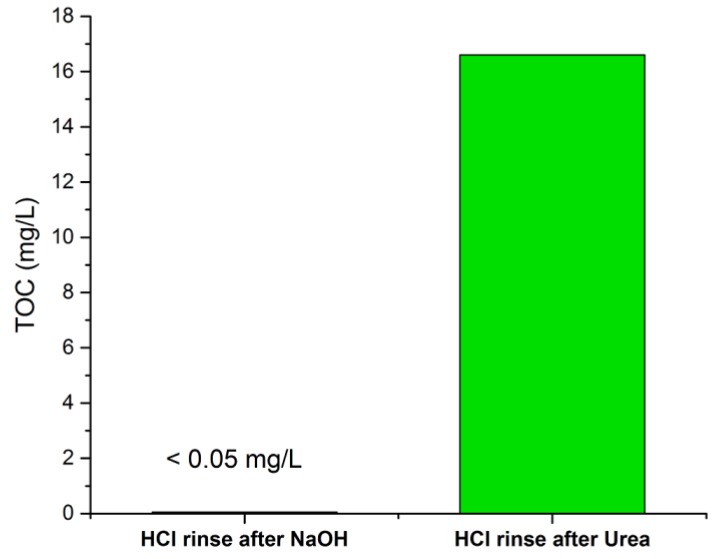
Total organic carbon content (mg/L) in the final stage acid rinse solutions after cleaning the membrane modules with NaOH and urea.

The amount of organic carbon was also measured in the final stage HCl rinse solution in the control and after cleaning the membrane modules. The TOC content was an order of magnitude greater in the HCl solution sampled after rinsing the membrane module cleaned with urea compared to the membrane module cleaned with NaOH (Figure 5).

### 3.4. Elemental Composition and Surface Morphology

**Figure 6:**
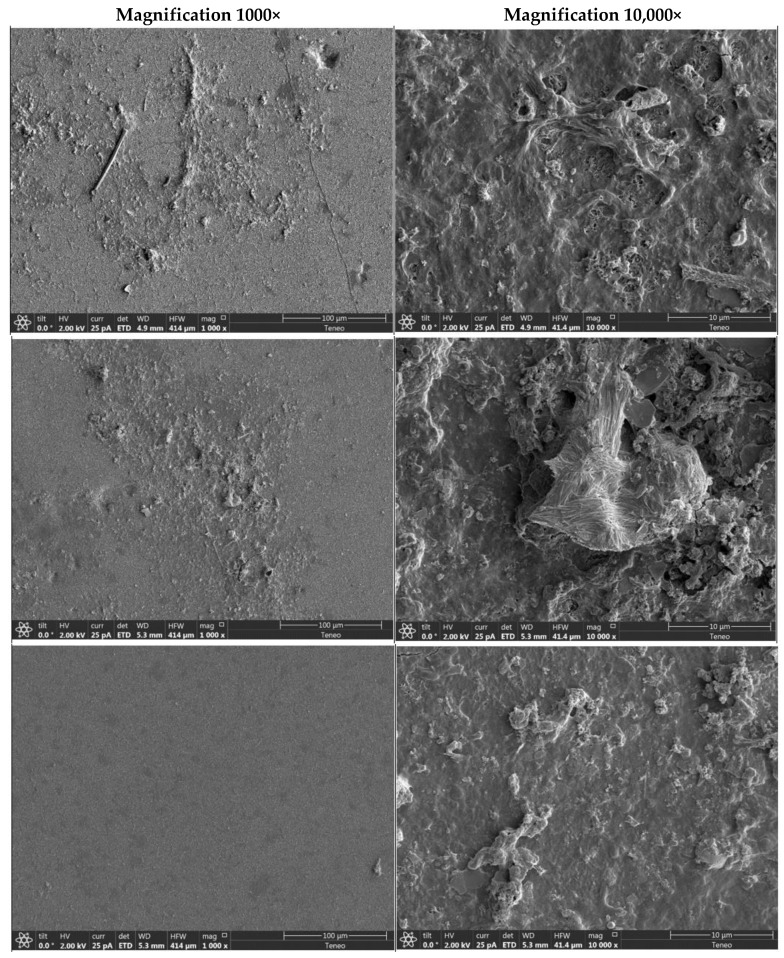
SEM images of the fouling surface on the uncleaned control membrane, and modules cleaned with NaOH + HCl and Urea + HCl.

SEM imaging (Figure 6) showed the occurrence of a surface fouling layer on the membranes, which could be easily differentiated from the surface expression of the polyamide membrane layer. The presence of colloids and biomass was apparent. Structures resembling bacterial cells and diatoms were observed, suggesting their presence in the fouling layer. SEM examination revealed no significant difference between the control (uncleaned) and reference (cleaned with NaOH + HCl) membrane samples in terms of the fouling layer removal efficiency. However, the screening of random locations of the membrane sample cleaned with Urea + HCl showed that the membrane surface was much cleaner than the reference and control membranes with a scarcely dispersed fouling layer.

The main elemental composition of the fouling layer was elicited using EDX analysis. Amongst the 13 elements detected in the EDX spectra (Figure S5), C, N, O and S were the most predominant elements (adding up to >90% weight), whereas Mg, Al, Si, P, K, Ca, Ti, Mn and Fe were only present in low concentrations (Table 3).

The EDX analysis suggests that biofouling and/or organic fouling plays the major role in the fouling of the membrane modules, as opposed to inorganic fouling and scaling. Proteins, polysaccharides and lipids are the main organic components encountered in the fouling of membranes and all of them contain carbon and oxygen. Nitrogen and trace amounts of sulphur are present in proteins and associated amino acids.

### 3.5. Molecular Composition

**Figure 7:**
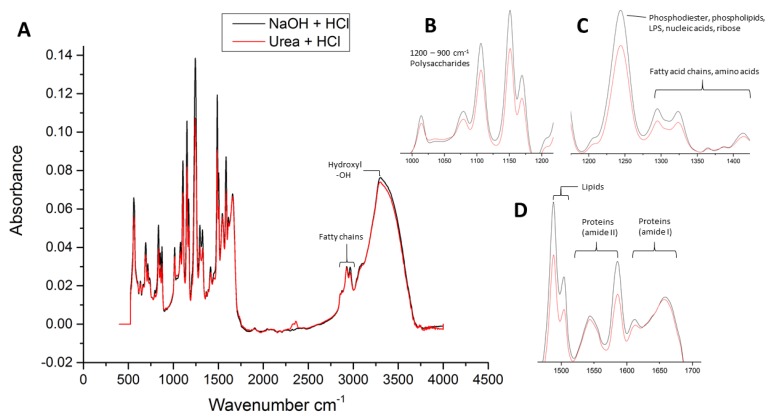
(**A**) Full FTIR spectra of the surface fouling layer on membrane modules cleaned with NaOH + HCl (**black**) and Urea + HCl (**red**). Zoomed in spectral regions of (**B**) polysaccharides, (**C**) phosphodiester, phospholipids, lipopolysaccharides, nucleic acids, ribose, fatty chains, amino acids, and (**D**) lipids and proteins.

The infrared spectra of the surface fouling layer deposited on the two cleaned membrane modules are presented in Figure 7. The peak assignments were allotted according to the literature [32,33]. In general, the membrane module cleaned with NaOH + HCl had a very similar spectrum to the module cleaned with Urea + HCl. However, the absorbance bands of all functional groups were lower in the membrane cleaned with urea, suggesting a higher solubilization of organic foulants by urea. The main fouling constituents included polysaccharides, proteins, fatty acid chains, lipids, nucleic acids and other compounds derived from humic substances. The broad region of absorption between 3400 and 3000 cm^−1^ is due to stretching of the O–H bond in hydroxyl functional groups. The sharper peaks at 2961 and 2925 cm^−1^ are due to the stretching fatty chains (ʋCH_3_, ʋCH_2_, υCH). The absorbance peaks of amide I (1684–1614 cm^−1^) and amide II (1587–1541 cm^−1^) suggest the presence of proteins and amino acids in the EPS. The polysaccharides region is shown in the spectral region of 1200–900 cm^−1^, corresponding to different stretching and bending vibrations (υC–O, υC–C, δC–O–H, δC–O–C). There are also clear peaks visible for lipids (1487 cm^−1^) and phosphodiester, phospholipids, lipopolysaccharides, nucleic acids and ribose compounds (1243 cm^−1^). In summary, the results of the FTIR spectra corroborate with the SEM–EDX analysis in terms of identifying organic foulants and biogenic materials as the major contributors to the fouling of spiral-wound membrane modules at the DECO water treatment plant. Moreover, the solubility of macromolecules in the biofilm is moderately enhanced by urea compared to conventional cleaning chemicals.

## 4. Discussion

Membrane cleaning is essential to restore the efficiency of filtration process operation in the water treatment industry. Routine cleaning of the RO membranes with acid and alkaline chemicals is the standard practice at the Evides DECO plant. This study examined the effect of replacing the typically used alkaline cleaning agent (sodium hydroxide) with a chaotropic agent (urea) in an effort to denature proteins and enhance the solubility of organic foulants. This research effort was the first of its kind in terms of employing urea for the chemical cleaning of spiral-wound RO membrane elements in a cleaning-in-place installation at a pilot-scale facility.

### 4.1. Membrane Regeneration

Confirming the previous lab-scale studies [23], the results of this pilot-scale study show that urea cleaning and the standard acid/alkali cleaning are equally effective with regards to restoring the normalized feed channel pressure drop. Both cleaning strategies fulfilled the aim of chemical cleaning i.e., to restore the feed channel pressure drop of the membrane element when it exceeds 10–15% of the start-up value. However, in terms of flux recovery, the performance of urea was better than NaOH cleaning. Urea cleaning increased the permeate flux by 5%, while the acid/alkali cleaning was not effective in improving the flux. This is probably due to the removal of surface adhered materials which are better dissolved by urea. The removal of the biomass from the spacer surface reduces the overall feed channel pressure drop, indirectly resulting in a higher flux. After cleaning with NaOH + HCl, the membrane permeability remained unchanged but the normalized pressure drop was restored by 13% (Figure 1). This may indicate a compaction of the fouling layer after NaOH cleaning. A similar finding was reported by Beyer et al., (2017). In this study [19], examining chemical cleaning at three full-scale RO plants, feed channel pressure drop improved by 10%, but permeability decreased by 5% and salt rejection remained unchanged, indicating a compaction of the fouling layer. It is also plausible that NaOH requires a longer contact time than urea to effectively restore membrane permeability.

### 4.2. Biofilm Solubilization and Removal

An autopsy and subsequent visual examination of the membrane elements revealed more fouling on the feed spacer surface than on the membrane. This finding is in agreement with previous studies which have shown that (i) initial biofouling deposition occurs on the surface of feed spacers and (ii) feed spacer biofouling effects overall performance more adversely than membrane biofouling [34,35,36,37]. Urea molecules are able to diffuse into the biofilm matrix and bacterial cells resulting in extracellular and intracellular swelling of the biofilm, eventually leading to osmotic lysis [22]. For this reason, biomass inactivation may be enhanced with urea-based chemical cleanings. With regards to biomass removal, the membrane module cleaned with urea + HCl visibly appeared cleaner than the module cleaned with the conventional chemicals. This was also observed during SEM and FTIR analysis, where urea cleaning produced a slightly better performance in terms of removing the biofilm. The membrane surface appeared cleaner post-urea-treatment and contained a lower concentration of organic compounds, suggesting an enhanced solubilization of the biofilm with urea treatment. Furthermore, total organic carbon analysis of the HCl rinse solution post cleaning the membrane modules revealed that urea is more efficient at solubilizing the surface fouling layer. The stability of the biofilm matrix is compromised when urea cleaning disrupts the hydrogen-bond network of the biofilm and creates a loose fouling layer, consequently enhancing the solubilization and removal of biomass.

### 4.3. Relevance of Urea Use

The use of wastewater effluent as feed water at the DECO plant increases the fouling tendency of membrane elements, particularly biological fouling due to the high biodegradable organic carbon content of membrane bioreactor permeate [9]. The analysis of the membrane surface morphology (SEM) and biofilm composition (EDX and FTIR) confirmed the presence of biological materials (biofouling) on the membrane modules. Cleaning of the membrane modules at the DECO plant with chemicals such as NaOH and urea is therefore a suitable cleaning strategy, since the reaction mechanism of both the cleaning agents results in the removal of organic fouling [19,38]. Preliminary laboratory studies confirmed the compatibility of urea with the polyamide membranes [23], where urea was not found to damage the polyamide layer of the membrane.

Biocides such as 2,2-dibromo-3-nitrilopropionamide (DBNPA) [39] and disinfectants such as monochloramine [9] can prevent or delay the formation of a biofilm, but they cannot prevent the deposition of organic foulants onto the membrane surface. Urea is capable of removing both biofilm from the feed spacer and organic fouling from the membrane surface, helping with lowering feed spacer pressure drop and increasing membrane permeability. Therefore, urea cleaning is an effective curative strategy for biofouling control. The application of urea treatment in a preventive mode requires further investigation. Perhaps a continuous or shock dosage of DBNPA can be incorporated to delay and reduce biofilm formation, along with urea cleaning every 3 days for preventive biofouling control [10]. However, given the toxicity of DBNPA, it must not be used in drinking water production. Similarly, during wastewater treatment, monochloramine application effectively controls biofouling in RO systems, but it is imperative to restrict the formation of disinfection by-products and decay of monochloramine.

### 4.4. Future Research

The fact that the membrane modules used in this study had been in operation for 2.5 years means there may have been some accumulation of an irreversible fouling layer in the membrane modules, making it difficult to fully restore the membrane permeability to the start-up values. Nevertheless, in order to increase the percentage of permeate flux recovery, the duration of cleaning should be extended from 1 h recirculation time to 3 or more hours in order to further disrupt the bonds between the foulants and the membrane surface, as well as weaken the chemical bonds within the EPS matrix. At the DECO plant, the NaOH and HCl cleaning phases last for >4 h, consisting of periods of high flow recirculation and soaking. The urea cleaning protocol must be optimized to include multiple stages of high flow recirculation and soaking, as practiced during CIP regimens in industry. Extended periods of recirculation with urea may reduce the frequency of routine chemical cleanings, as already implemented by the DECO plant, to maintain filtration process productivity. The introduction of a surfactant in the cleaning protocol could result in greater cleaning efficiency by reducing the surface tension and increasing the solubility of foulants. Recent studies have demonstrated a greater cleaning efficiency with combined sequential cleaning with NaOH and sodium dodecyl sulfate (SDS), compared to the use of NaOH only [40,41].

In contrast with the single cleaning cycle conducted in this study, conducting multiple urea cleaning cycles may be interesting to examine the impact on biofilm structure and composition. The effectiveness of urea cleaning to restore membrane performance during long-term membrane operation should be studied. Routine chemical cleaning with urea may be optimized such that urea is recycled from the waste solution after cleaning the membrane modules. Initial experiments have already been carried out successfully to recover the urea using eutectic freeze crystallization. The potential of recycling urea from the waste cleaning solution could result in significant reductions in the amount of chemical waste and the costs associated with cleaning, such as the purchase and transport of chemicals and the treatment of chemical waste. Suitable urea reuse methods must be applied in order to encourage the eco-friendly use of urea over the chemically wasteful conventional cleaning strategies.

## 5. Conclusions

The chemical cleaning efficiency of urea was compared with the conventionally applied cleaning solutions (sodium hydroxide and hydrochloric acid) for spiral-wound RO membrane elements taken from a full-scale installation and cleaned at a pilot-scale facility in the Netherlands. Based on the results of this study, it can be concluded that:

Biofouling plays the major role in the fouling of the membrane elements at the DECO plant.

Urea cleaning is as effective as the conventional chemical cleaning in terms of restoring the normalized feed channel pressure drop, and more effective in terms of (i) restoring membrane permeability; (ii) solubilizing organic foulants and (iii) removing the surface fouling layer.

## Figures and Tables

**Table 1:** Chemical cleaning strategies applied to each membrane element. Performance data of all three membrane modules was recorded before, during and after cleaning.

Module	Code	Cleaning Protocol	Comment
1	Control	None	Control module. No cleaning
2	NaOH + HCl	(i) NaOH (pH 12, 35 °C) recirculated for 1 h (ii) HCl (pH 1, room temperature) (~18 °C) recirculated for 30 min	Reference module cleaned with conventional alkali/acid solutions as applied by DOW and Evides
3	Urea + HCl	(i) CO(NH_2_)_2_ (1340 g/L, 35 °C) recirculated for 1 h (ii) HCl (pH 1, room temperature) (~18 °C) recirculated for 30 min	NaOH replaced by saturated urea solution

**Table 2:** Membrane performance parameters before, during and after cleaning.

Performance Parameters	Cleaning with NaOH + HCl			Cleaning with Urea + HCl		
	Initial	After NaOH	After HCl	Initial	After Urea	After HCl
Normalized Feed Channel Pressure Drop (mbar)	208	202	181	133	173	115
Normalized Flux (Lm^−2^ h^−1^)	29.07	29.98	29.07	29.68	29.98	31.19

**Table 3:** Comparison of elemental composition (% weight) of the fouling layer as determined by energy dispersive X-ray (EDX) analysis.

Element	C	N	O	Mg	Al	Si	P	S	K	Ca	Ti	Mn	Fe
Control	66.26	6.78	18.77	0.28	0.41	0.34	0.55	4.31	0.15	0.29	0.21	0.53	1.12
NaOH + HCl	62.44	3.34	21.98	0.30	0.60	0.79	0.59	5.96	0.21	0.27	0.18	0.44	2.91
Urea + HCl	59.31	6.88	24.88	0.37	0.68	0.91	0.61	5.02	0.23	0.66	0.26	0.47	2.42

## Supplementary Materials

The following are available online at https://www.mdpi.com/2077-0375/9/9/117/s1, Figure S1: Automated RO pilot installation equipped with two single 8-inch module pressure vessels, designed to measure key performance indicators in real time. Figure S2: Fouling on the inlet and outlet ends of spiral-wound membrane modules after 2.5 years of operation at the DECO water treatment plant. Figure S3: Unwound control membrane module (uncleaned) after membrane autopsy. Visual observations showed that the bulk of the fouling is present on (A) the inlet side rather than the middle or outlet of the module, and (B) the spacer surface rather than the membrane surface. Figure S4: Visual comparison of the membrane/spacer surface of cleaned membrane modules. Figure S5: Energy dispersive x-ray spectra of (A) Control module uncleaned, (B) Reference module cleaned with NaOH + HCl and (C) Test module cleaned with Urea + HCl. The EDX spectra show that C, N, O and S dominate the elemental composition, thereby confirming the presence of organic fouling and biofouling. Scaling or inorganic fouling may be very minor contributors to overall fouling of the membrane modules since elements like Al, Ca and Fe were present only in trace concentrations.
