# Supplementary material for: Pilot-Scale Assessment of Urea as a Chemical Cleaning Agent for Biofouling Control in Spiral-Wound Reverse Osmosis Membrane Elements
Source: Membranes (Basel). 2019 Sep 6;9(9):117. doi: 10.3390/membranes9090117 (PMC6780893; doi:10.3390/membranes9090117)
Supplement: Supplementary file 1 [file membranes-09-00117-s001.pdf]

Pilot-Scale Assessment of Urea as a Chemical Cleaning Agent for Biofouling Control in Spiral-Wound Reverse Osmosis Membrane Elements

Huma Sanawar [1,*], Szilárd S. Bucs [1], Martin A. Pot [2], Jure Zlopasa [3], Nadia M. Farhat [1], Geert-Jan Witkamp [1], Joop C. Kruithof [4], Mark C.M. van Loosdrecht [3] and Johannes S. Vrouwenvelder. [1,3]

[1] King Abdullah University of Science and Technology (KAUST), Water Desalination and Reuse Center (WDRC), Division of Biological and Environmental Science and Engineering (BESE), Thuwal 23955-6900, Saudi Arabia

[2] Evides Industriewater BV, Process & Technology Department, P.O. Box 4472, 3006 AL Rotterdam, the Netherlands

[3] Department of Biotechnology, Faculty of Applied Sciences, Delft University of Technology, Van der Maasweg 9, 2629 HZ Delft, the Netherlands

[4] Wetsus, European Centre of Excellence for Sustainable Water Technology, Oostergoweg 9, 8911 MA, Leeuwarden, the Netherlands

* Correspondence: huma.sanawar@kaust.edu.sa

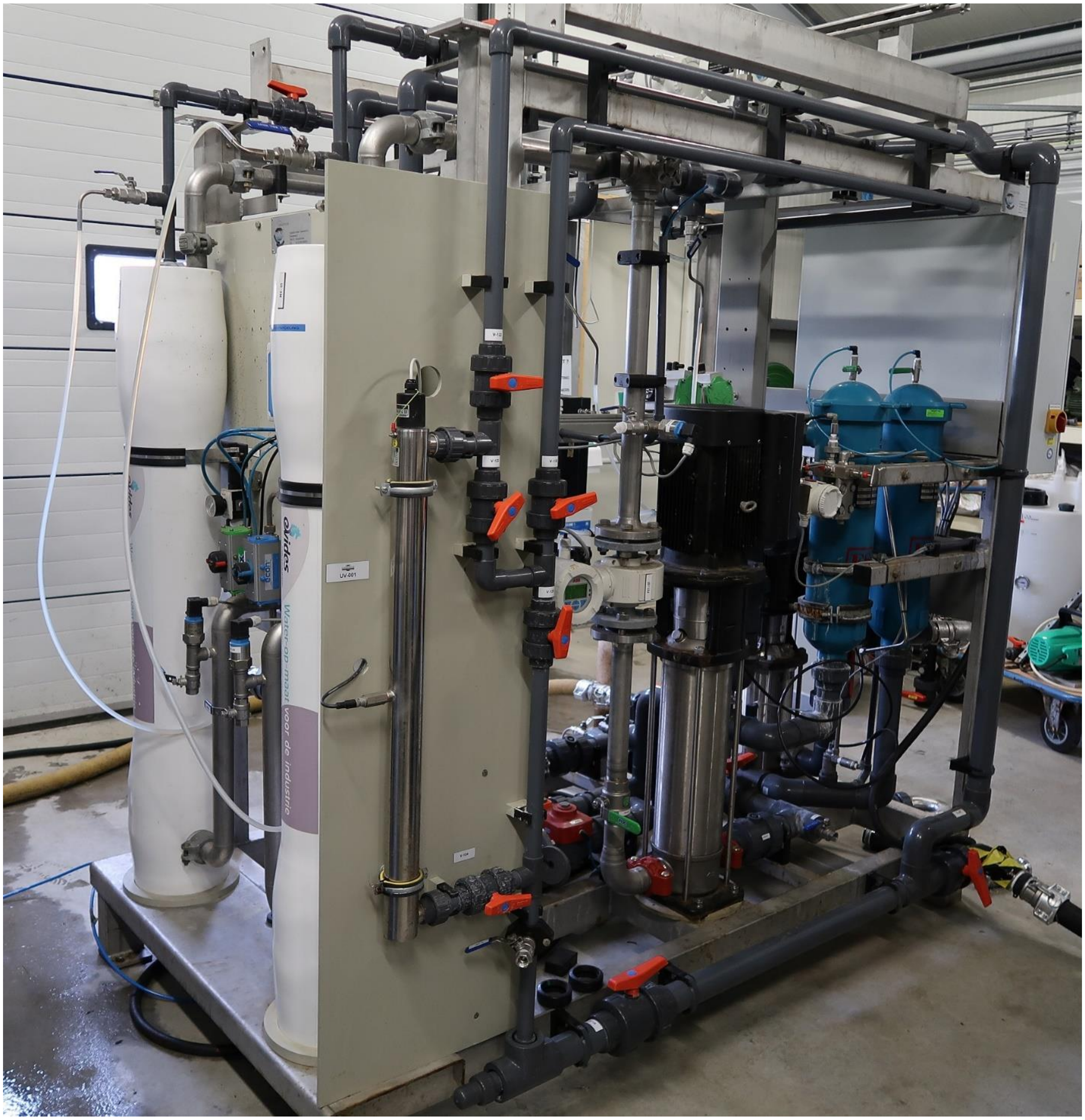

Figure S1: Automated RO pilot installation equipped with two single 8-inch module pressure vessels, designed to measure key performance indicators in real time.

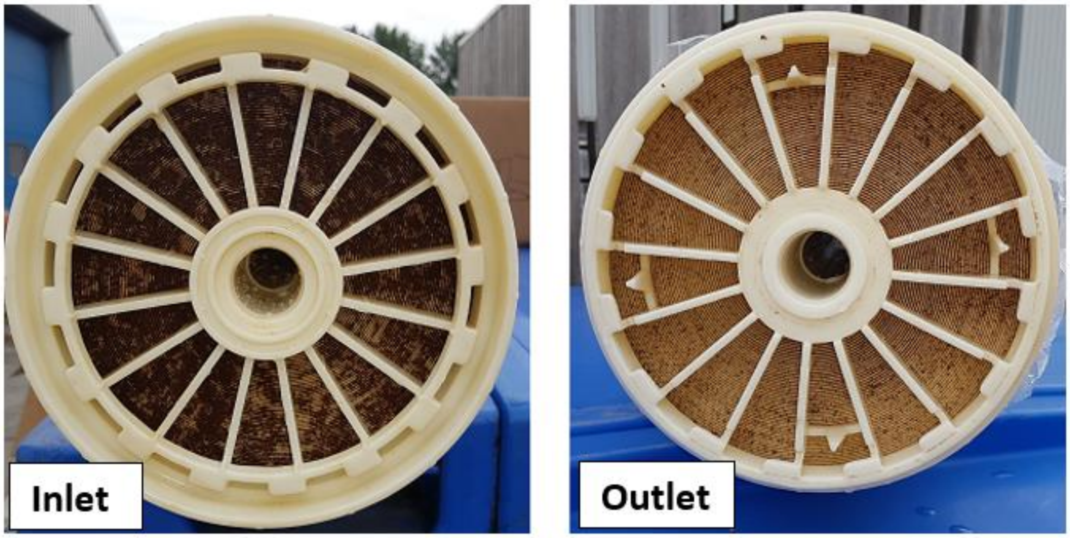

Figure S2: Fouling on the inlet and outlet ends of spiral-wound membrane modules after 2.5 years of operation at the DECO water treatment plant.

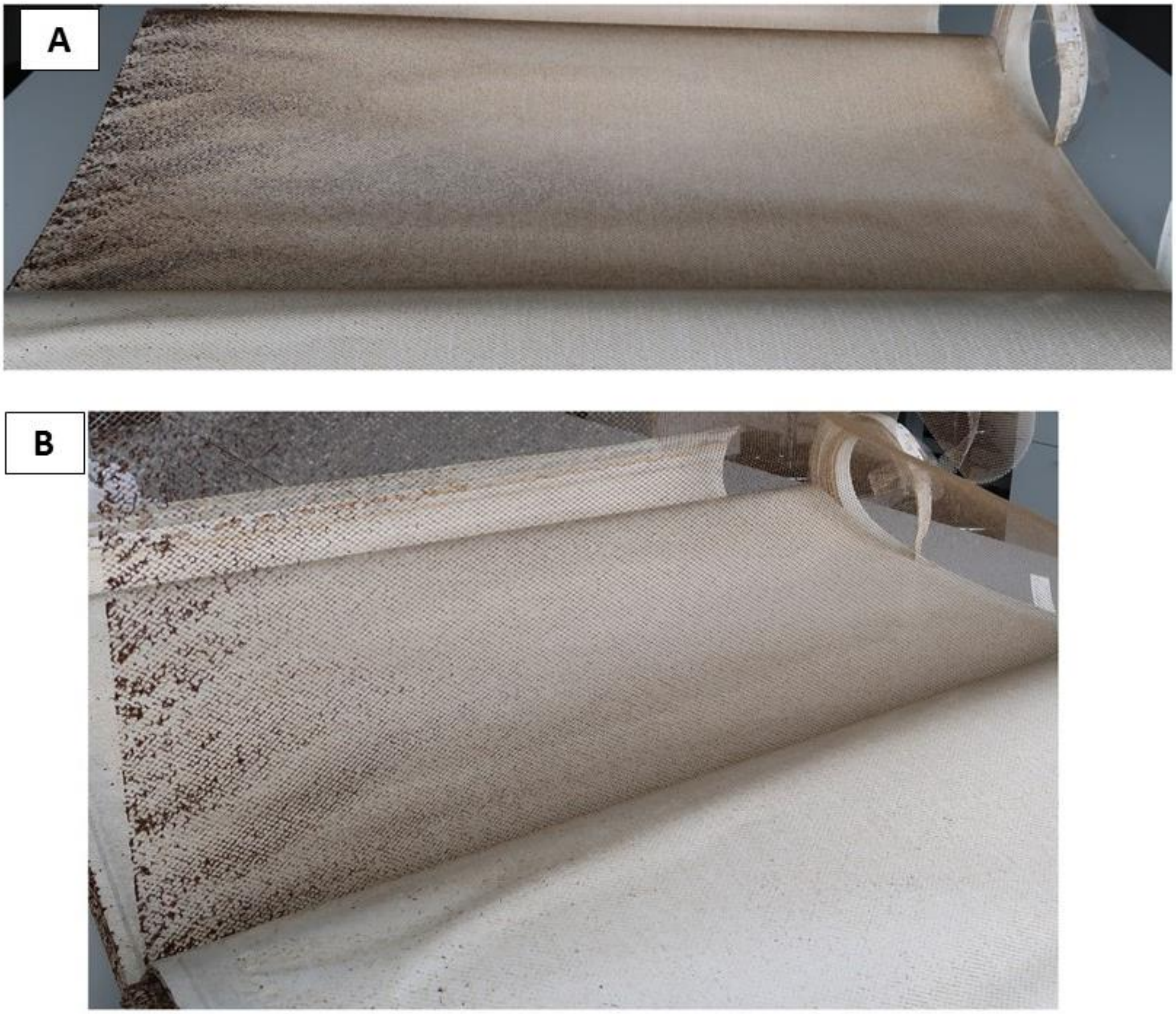

Figure S3: Unwound control membrane module (uncleaned) after membrane autopsy. Visual observations showed that the bulk of the fouling is present on (**A**) the inlet side rather than the middle or outlet of the module, and (**B**) the spacer surface rather than the membrane surface.

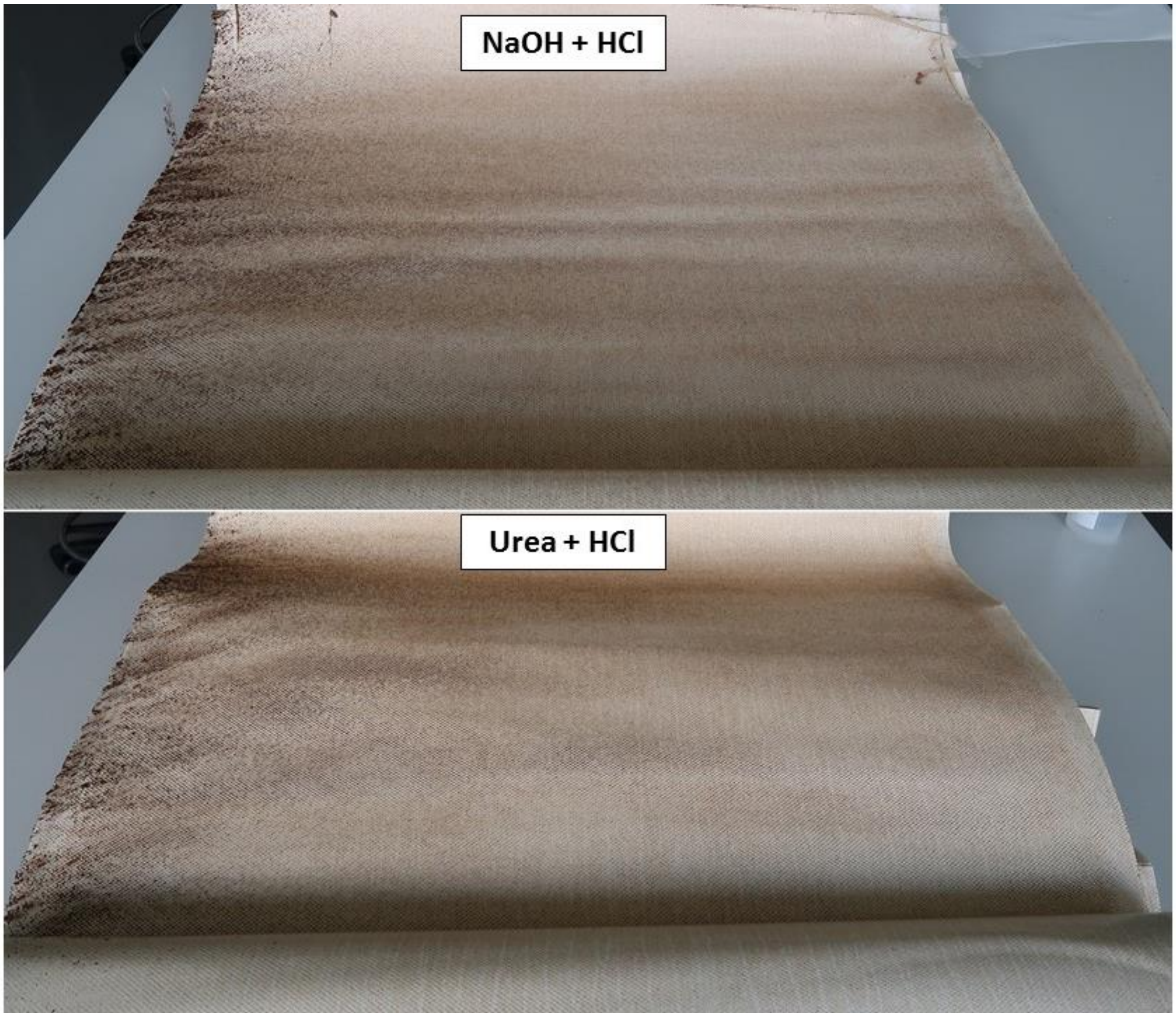

Figure S4: Visual comparison of the membrane/spacer surface of cleaned membrane modules.

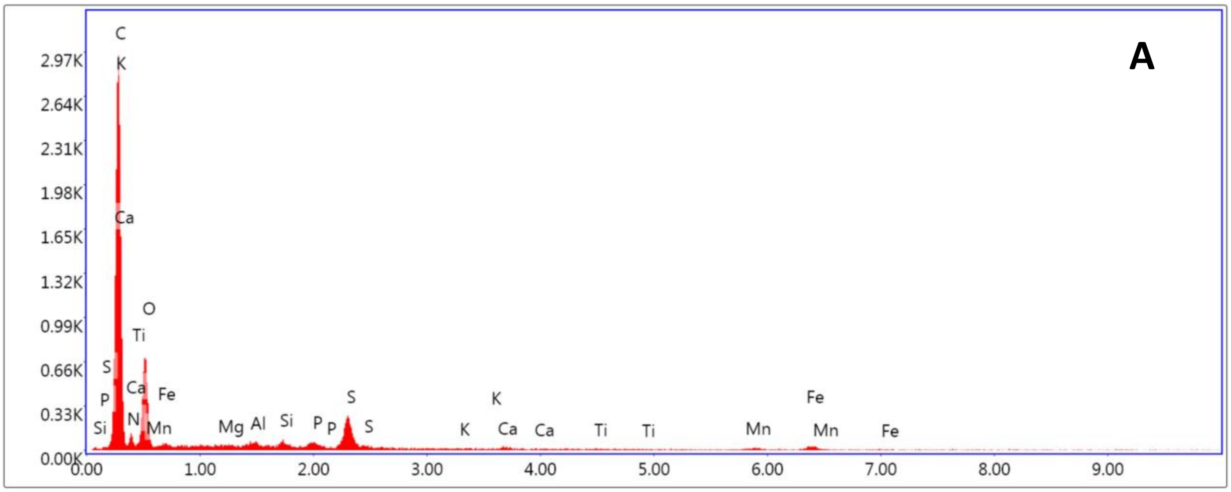

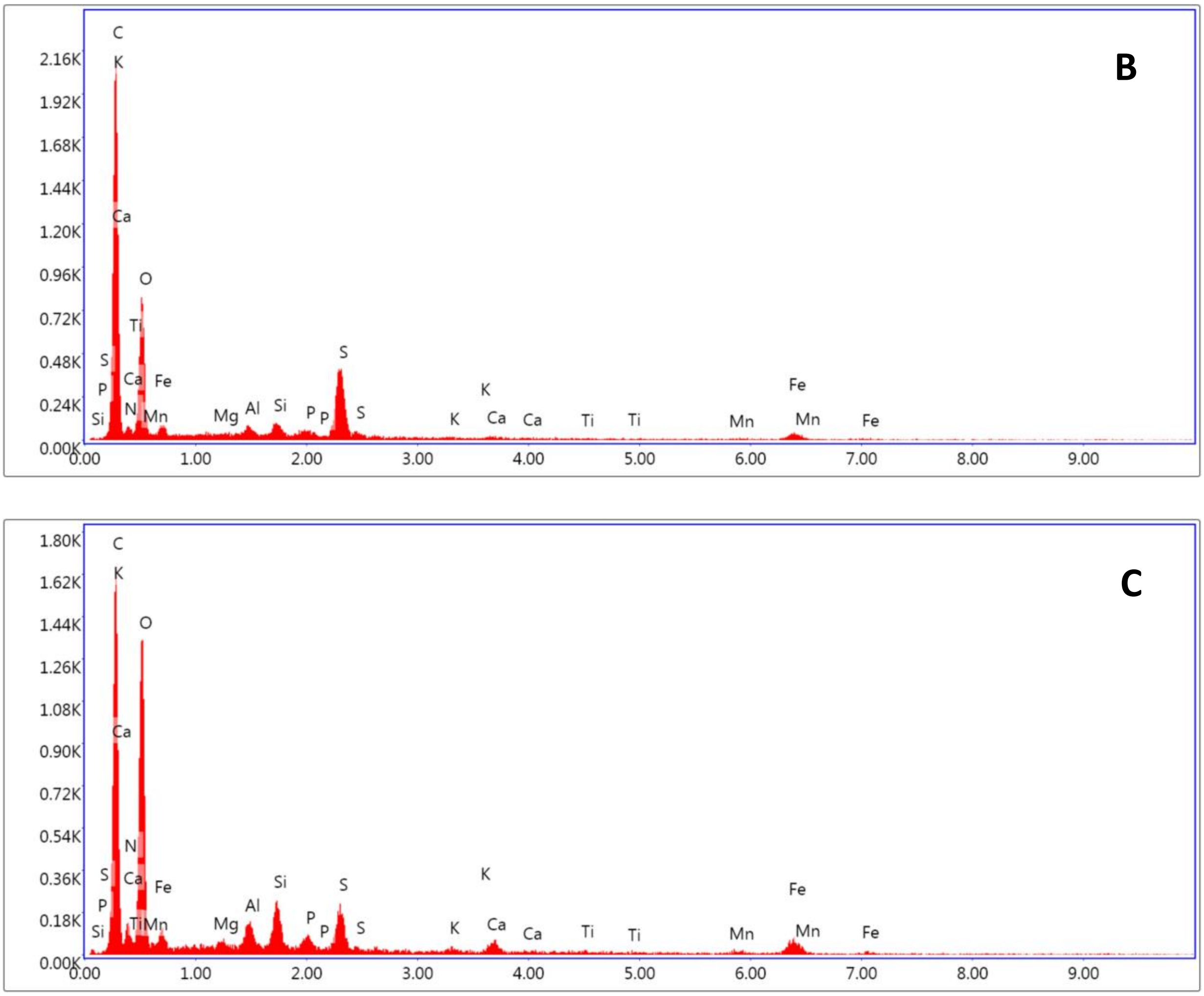

Figure S5: Energy dispersive x-ray spectra of (**A**) Control module uncleaned, (**B**) Reference module cleaned with NaOH + HCl and (**C**) Test module cleaned with Urea + HCl. The EDX spectra show that C, N, O and S dominate the elemental composition, thereby confirming the presence of organic fouling and biofouling. Scaling or inorganic fouling may be very minor contributors to overall fouling of the membrane modules since elements like Al, Ca and Fe were present only in trace concentrations.
